# Anxious driving behavior among taxi drivers

**DOI:** 10.1192/j.eurpsy.2021.488

**Published:** 2021-08-13

**Authors:** M. Bejar, N. Regaieg, D. Gdoura, J. Aloulou, O. Amami

**Affiliations:** 1 Psychiatry B Departement, Universal Hospital Hedi chaker, Sfax, Sfax, Tunisia; 2 Psychiatry “a” Department, Hedi Chaker UHC, Sfax, Tunisia, Sfax, Tunisia; 3 Psychiatry (b), Hedi Chaker University hospital, sfax, Tunisia; 4 Psychiatry (b), Psychiatry (B), Hedi Chaker University hospital, sfax, Tunisia; 5 Psychiatry (b), PsHedi Chaker University hospital, sfax, Tunisia, Sfax, Tunisia

**Keywords:** Anxiety, performance deficits, exaggerated caution, Taxi drivers

## Abstract

**Introduction:**

The data suggest that anxious drivers may engage in problem behaviors that expose them and others to an increased risk of negative traffic events.

**Objectives:**

To study the problematic behavior taxi drivers related to anxiety in three areas exaggerated safety/caution, performance deficits, and hostile/aggressive behaviors and to determine the factors who are associated with them.

**Methods:**

This is a cross-sectional descriptive and analytical study of 58 taxi drivers in the city of Sfax, Tunisia. We used an anonymous questionnaire that included a socio-demographic fact sheet, and a driver behavior rating scale: Driver Behavior Survey (DBS) with 21 items.

**Results:**

The mean age of the drivers was 40.8 ± 10.2 years. The sex ratio was 0.98. 75.9% were married. 6.9% lived alone. 53.4% were smokers and 25.9% drank alcohol. Coffee and tea consumption were 59% and 33% respectively. 67% had a pathological personal history, including osteoarticular pathologies. 17.2% had a history of serious accidents. The behavior related to anxiety among taxi drivers was 74.66 ± 13.35. The hostile behavior was 18.88 ± 8, the exaggerated safety behavior was 38.31 ± 7.3 and the deficit performance related to anxiety was 17.47 ± 7.1. The problematic behavior in our population was significantly associated with lifestyle alone, coffee consumption and with serious accidents.

**Conclusions:**

The results of our study identified some risk factors that could lead to poorly adaptive driving behaviors among Taxi drivers. These elements reinforce us in the idea that this population requires special care with a meeting with the doctor.

